# Preschoolers’ Attitudes, School Motivation, and Executive Functions in the Context of Various Types of Kindergarten

**DOI:** 10.3389/fpsyg.2022.823980

**Published:** 2022-03-03

**Authors:** Jana Kvintova, Lucie Kremenkova, Roman Cuberek, Jitka Petrova, Iva Stuchlikova, Simona Dobesova-Cakirpaloglu, Michaela Pugnerova, Kristyna Balatova, Sona Lemrova, Miluse Viteckova, Irena Plevova

**Affiliations:** ^1^Department of Psychology and Abnormal Psychology, Faculty of Education, Palacký University Olomouc, Olomouc, Czechia; ^2^Faculty of Physical Culture, Institute of Active Lifestyle, Palacký University Olomouc, Olomouc, Czechia; ^3^Faculty of Education, Institute of Education and Social Studies, Palacký University Olomouc, Olomouc, Czechia; ^4^Department of Psychology, Faculty of Education, University of South Bohemia in České Budějovice, České Budějovice, Czechia; ^5^Department of Primary and Pre-Primary Education, Faculty of Education, University of South Bohemia in České Budějovice, České Budějovice, Czechia

**Keywords:** kindergarten, attitudes, motivation, executive functions, school achievement

## Abstract

European policy has seen a number of changes and innovations in the field of early childhood preschool education over the last decade, which have been reflected in various forms in the policies of individual EU countries. Within the Czech preschool policy, certain innovations and approaches have been implemented in the field of early children education, such as the introduction of compulsory preschool education before entering primary school from 2017, emphasis on inclusive education, equal conditions in education and enabling state-supported diversity in the education concepts of kindergartens. The aim of our study was to assess the influence of various preschool education systems in the Czech Republic in the context of psychological variables reflecting selected children’s outcomes which may contribute to future school achievement. The monitored variables were the attitudes, motivations and executive functions of children in the last year of preschool education. A comparison was made between the traditional preschool education program and the so-called alternative types of preschool education, such as Montessori, Waldorf and religious schools. The total sample was divided into four subgroups, namely a group of children attending traditional kindergartens (731, 84.9%), religious (65, 7.5%), Montessori (35, 4.1%), and Waldorf (30, 3.5%) kindergartens. To determine empirical data, the following research methods were used: Attitude Questionnaire, School Performance Motivation Scale, and Behavior Rating Inventory of Executive Function (BRIEF). The results of our survey show the fact that the type of kindergarten attended has a significant effect on the child’s level of school performance motivation, attitudes toward school as well as executive functions. Significant differences were found between the different types of kindergartens attended in the monitored variables.

## Introduction

The policy discourse in EU countries around early childhood education has changed significantly over the past twenty years, from childcare to education, from childcare as a policy mechanism to increase women’s employment to the focus on children’s outcomes. Thus, the result was an increased acknowledgment of the role of preschool education in “laying the foundations for learning at school,” and also emphasizing the good quality, effectiveness and inclusiveness of early education as being beneficial for all children. Countries within the EU have adopted such a policy approach and made changes in congruence with the recommendations of the EU Council. However, there are still discrepancies between countries and/or regions within the European landscape influenced by different factors, such as specifics of the cultural and social context, different educational systems, parameters of practice for preschools, different teacher education quality, etc. (Ou et al., 2004; Yoshikawa et al., 2013; McCoy et al., 2017; Council of the European Union, 2019; Alexiadou and Altmann, 2020). In the context of the Czech preschool policy, innovations and approaches were implemented in early children education including for example the introduction of compulsory 1-year preschool education before enrollment in elementary school (from 2017), emphasis on inclusive education and equal conditions in education as well as state-supported diversity in kindergarten education systems. However, there is a lack of knowledge about the influence of kindergarten including different kindergarten education systems on later school achievement, not only in the Czech Republic but also in other countries.

In the Czech Republic there are several state-supported preschool education concepts, with all types of kindergartens having to fulfill the expected outcomes of the general state-required curriculum as part of their educational activity. The way of their achievement is based on the basic principles of education concepts. The majority of kindergartens follow the traditional preschool educational system (about 85%) while the minority (about 15%) consist of the so-called “schools with alternative educational programs” (further only “alternative schools”), such as Montessori, Waldorf, and religious schools. The choice of these three alternative preschool programs was motivated by the fact that there are no other alternative educational programs in the Czech Republic.

### Montessori Educational System

Montessori’s education system sees the child as an active being capable of concentration, independent work and discovery (Montessori, 2011) who can independently build his/her individuality provided that he/she has adequate guidance (prepared adult), pedagogically prepared environment as well as unique learning materials that emphasize the teaching of practical life skills, sensory development, literacy, and math competence as well as cultural appreciation (Petrash, 2002; Montessori, 2011; Phillips-Silver and Daza, 2018). Montessori education has been in existence for over 100 years (Lillard, 2012); however, studies reflecting the educational impact on child development in comparison with traditional educational programs are rare. Studies carried out so far have brought contradictory results. Some research studies suggest an improvement in learning and development of children in Montessori education compared with other educational programs (Lillard and Else-Quest, 2006; Glenberg et al., 2007; Besançon et al., 2015), for example in gross and fine motor skills, language and social behavior as well as in areas that may affect intellectual development (He et al., 2009) or executive functions (Lillard, 2012). A longitudinal study by Lillard (Lillard et al., 2017) revealed better results among children from Montessori preschool education in academic achievement, social understanding, mastery orientation as well as executive functions, while in the area of social problem solving or creativity no significant differences were found. An interesting add-on effect of the Montessori program was the reduction of the effect of the income gap on children’s academic achievement (Lillard et al., 2017). On the contrary, for example a study on school achievement in connection with the level of creativity did not suggest any significant differences between children from Montessori schools and children from traditional schools (Cox and Rowlands, 2000). With regard to school-aged children’s long-term cognitive skills as the result of the conducted research, it was suggested that there was no clear evidence in favor of Montessori programs (Lopata et al., 2005). Ansari and Winsler (2014) indicate that all children in their study, regardless of curriculum, demonstrated gains across pre-academic, socio-emotional, and behavioral skills throughout their preschool year, however those enrolled in Montessori programs did not exhibit greater gains compared to children enrolled in traditional preschool programs. These inconsistent results may be explained by different effects, including for example methodological weaknesses, small sample size, non-existence of a control group, variations in Montessori implementation fidelity, etc. (Lopata et al., 2005; He et al., 2009; Lillard, 2012); at the same time, this increases the need for further research, for example in the form of randomly controlled studies.

### Waldorf Educational System

Waldorf educational system was developed in the early twentieth century and is based on R. Steiner’s concept (Petrash, 2002; Petlák, 2004). Waldorf education emphasizes the focus on a child’s freedom and holistic child development (Aljabreen, 2020). Education is fully subject to the development and instigation of the child’s activity, interests and needs (Průcha, 2004), and personal freedom to its greatest potential (Edmunds, 2012). Lessons are created according to the children’s developmental stages, maximizing their potential to learn based on critical development periods that they are facing (Nordlund, 2013). The role of the teacher is to provide guidance for the child and acts as an artistic director supporting the child’s learning in the areas of arts: goodness, beauty, and truth (Edwards, 2002; Aljabreen, 2020) as well as a promotor to help the child to build practical skills, mainly through game (Petlák, 2004). While research on the former alternative program is rare, in the case of Waldorf education the situation is even more limited. This may be caused by a lack of interest in conducting comparative studies. In this context, it should be noted that in the past Waldorf pedagogy was considered as controversial; however, during the last nearly two decades it has changed its attitude to educational sciences and teacher training institutions have become more academic as a result of the Bologna process (Paschen, 2014).

Former learners have a generally positive assessment of the Waldorf education model; they emphasize the attainment of competences such as a positive attitude to life, confidence in their own strength/ability, independence as well as the ability to adapt to life conditions (Ionova, 2013). From a pedagogical perspective they appreciate benefits including deeper retention of learned material as well as the ability to use artistic processes to learn (Nordlund, 2013). However, their performance outcomes in standardized test methods are mixed. According to a comparative study including pupils from traditional models, Waldorf learners underperformed up to the second grade, subsequently their performance equalized or even better results were reported in some subjects (Oberman, 2007). These results were confirmed by another study in which better results in standardized tests were achieved by pupils who started Waldorf education in grade 3 compared with those who had already begun in preschool age (Ogletree, 1996). Another study suggests better results of successful Waldorf learners in admission exams to German universities, on average by 20–30% (Ionova, 2013). Whether the worse results at the beginning of education are caused by the fact that pupils are not educated with the intent of passing exams (Ogletree, 1996), that there is a lack of performance requirements and assessment (Rawson, 2015) as well as a lack of technological integration, or the reasons are different, there is a need for further research. Montessori and Waldorf education remain two of the most popular models for alternative early childhood education; each of these approaches has developed globally (Aljabreen, 2020).

### Religious Kindergarten

The last type of kindergarten in our study is religious kindergarten, which is one of the types of alternative schools emphasizing religious education and morality in the spirit of a particular confession (Christian, Jewish, etc.) (Průcha et al., 2003). In the Czech Republic, religious schools can only be established by churches and religious communities recognized by the state. Their alternative nature does not lie only in the type of the school authority but mainly in pedagogical and didactic specificities—especially the field of the curriculum and ideological principles of educational activity (for example principles of Christianity, Christian ethics, cultural traditions, etc.) (Průcha, 2004). In the Czech Republic, the predominant religion in these types of schools is Christianity. The daily regime of religious kindergartens does not differ significantly from traditional kindergartens. The only exception relates to regular prayers during the day and various events, or celebrations organized throughout the year in connection with faith (Easter, Christmas, etc.). This type of kindergarten is often attended by children from non-religious families who appreciate educational procedures based on the principles and values of Christian thinking and behavior.

### Traditional Kindergarten

Pre-school education in the Czech Republic is governed by the curricular document that is called Framework Education Programme for Preschool Education which defines the content, conditions and rules for institutional education of preschool children. Regarding the fact that this is only a framework document, individual kindergartens have relative freedom in the development of their own school educational programs, the purpose of which is to reflect the circumstances of individual educational institutions. Despite this fact, traditional kindergartens have some common characteristics based on the requirements of the curriculum and the historical context of Czech preschool education. In traditional kindergartens, the content and offer of education (activities that pupils perform) is selected, presented and controlled mostly by the teacher. During the whole day, children are led by the teacher who primarily uses the mass form of organization in which knowledge is transmitted to children and the whole educational process is guided by the rhythm set by the teacher. This is due to the fact that traditional kindergartens are attended by 24–28 children per class, which makes it impossible for the teacher to approach each child individually. Therefore, teachers in traditional kindergartens are mostly driven to choose an achievable educational content and feasible methods. This is the greatest difference between traditional schools and Montessori and Waldorf schools. Another difference can be seen in their different material conditions. Classrooms in traditional kindergartens are divided into playing/working and sleeping parts and so the space is not structured according to the educational offer. Another difference is in the toys and aids which are usually not specific in terms of their didactic purpose and their location in the classroom does not correspond with clear rules (arrangement according to educational content, materials, etc.). As suggested by the above, traditional kindergartens differ from alternative kindergartens in the methods, forms and organization of the educational process.

### Psychological Predictors of School Achievement

School achievement, or future school achievement, is a construct the beginnings of which reach pre-primary education, and which focuses on the development of children’s attitudes to the educational institution but also their working and social habits. Also, they get the first opportunity to learn about and use their abilities and compare them with their classmates’. This initial experience can have in many aspects a relatively significant formative potential for the upcoming school attendance (Dumas and Lefranc, 2010; Pholphirul, 2017; Puhan et al., 2019). For preschool children and younger school-aged children the experience of success is of extreme importance because it helps develop healthy self-esteem, motivation as well as positive attitudes to specific activities (in this case to school and school work) (Széll, 2013). This was also confirmed by E. Erikson’s concept (Guinee, 1998) who in the period of initiative emphasizes the importance of developing the child’s personality in terms of its focus and self-determination. In other words, preschool children initiate their own activity and try to succeed.

School achievement is subject to a number of factors one of which is school motivation (Broussard, 2004), effort to achieve goals (success) or to avoid failure (cf. for example (Brdar et al., 2006), although in the case of goal-oriented behavior, the relationship with school achievement was mediated by coping strategies). The theory of performance motivation is based on the idea of the independence of need to achieve successful performance/school success and the need to avoid failure. The ratio of these needs influences students’ attitudes and behavior in performance situations and thus their learning effectiveness. Based on the knowledge of the intensity and ratio of motivational needs of pupils, it is possible to choose the optimal difficulty of tasks for individual pupils and at the same time to optimize the teacher’s work with motivation in the classroom (Hrabal and Pavelková, 2011).

The relationship between attitudes to school (or education) and school achievement was repeatedly confirmed by numerous studies (Moè et al., 2009; Carbonero et al., 2015; Veresova and Mala, 2016), although they mostly included students during prepuberty, puberty or adolescence, or younger school-aged children (Daviran, 2014; Turner and Pale, 2019). It should be emphasized that these attitudes are shaped already on the basis of children’s initial school experience (Daviran, 2014). Therefore, this issue needs to be addressed from the beginning of school attendance (including pre-primary and primary education). A more comprehensive approach to this issue was offered by Stephanou (2014) who confirmed a slightly positive effect of feelings (emotional component of attitudes) of children in kindergarten regarding their relationships with teachers on their beliefs about their competences, motivation to learn (intrinsic motivation, learning goals) as well as school achievement in mathematics.

Executive function is a construct that refers to interrelated neuropsychological functions and is involved in guiding, directing, regulating, and managing cognitive, behavioral, and emotional functions that are important to academic and life success (St John et al., 2018). These processes are developed in childhood; especially in preschool children attention is focused on self-regulation, impulse control, working memory and mental flexibility. However, there are also studies suggesting that preschool children also show planning, organization and decision-making abilities (Welsh et al., 1991). Many studies confirm a close relationship between school achievement and executive functions (Bull et al., 2008; Clark et al., 2010; Miller and Hinshaw, 2010; Willoughby et al., 2011), often in relation to mathematics and reading (St Clair-Thompson and Gathercole, 2006; Blair and Razza, 2007; van der Sluis et al., 2007; Best et al., 2011) in children of different ages, even in connection with the absence/presence of specific learning disorders (Best et al., 2009). Executive processes are often associated with emotional, behavioral and social functioning (Kochanska et al., 2000; Blair, 2002; Espy et al., 2011; Schoemaker et al., 2012) and provide an important biological basis for cognitive as well as socio-emotional school readiness (Barkley, 2001; Blair, 2002).

It is evident that the period of preschool education has a significant impact on the formation of the child’s personality and on his/her future, not only school achievement. However, despite the availability of numerous studies addressing this issue in recent decades, there is still a lack of studies covering the impact of the different forms of preschool education on child development and covering multiple types of kindergartens at the same time. To address this gap, we examined several key predictors (such as school performance motivation, attitudes, and executive functions) of school achievement in a cohort of children from traditional and three different alternative types of kindergartens.

The importance of this study lies in several reasons. First, the preschool environment and education represent the first major non-familial influence that shapes the child’s personality humanly, socially, cognitively, and performance-wise (and in the future, vocationally) (Bissoli, 2014; McCoy et al., 2017; Fessler and Schneebaum, 2019; Kajiume and Kobayashi, 2019). Second, preschool education has a non-negligible influence on future school achievement (Cryan et al., 1992; Schluter et al., 2020). Third, preschool education can counterbalance the negative developmental influences in the child’s environment and thus positively affect his or her future successful functioning in society. Fourth, although both the effect of preschool education on later school achievement and the effect of alternative forms of education have been extensively researched for several decades, there are not many studies available that include more than one type of preschool and thus allow direct comparisons between them. And fifth, despite the indicated importance of preschool education, there is still a lack of valid evidence (geographically or subject-wise) on the impact of the different forms of preschool education on the child’s future development, specifically in the context of the effect of alternative kindergarten approaches.

## Materials and Methods

### Participants and Procedures

The selection of the sample of kindergartens was based on two criteria. The first was the proportional representation of children from each type of kindergarten corresponding to the national proportional representation of these educational programs in the Czech Republic. The second was that each kindergarten had an associated primary school, as this study is part of a project examining the longitudinal impact of selected variables on later school achievement in the early years of primary school.

First, the required file size was calculated. To get the total population size, data from the Ministry of Education, Youth and Sports on the number of children enrolled in kindergarten by age for the academic year 2019/2020 were used (Ministry of Education, Youth and Sports [MEYS], 2019). For this study, the total population was calculated as the number of children aged 5 years and above (which mainly represents the last year of kindergarten before entering school). The resulting population was 12,717 children. A minimum sample size of 373 was derived from this number by requiring a 95% confidence level and 5% margin of error. Considering the expected uneven distribution of children across the different types of kindergartens and accounting for the risk of some responses being dropped during quality control, the required sample size was set at 2.5 times the minimum sample size, i.e., approximately 900 children.

The recruitment of kindergartens into the study was initially based on random sampling. From a list of eligible kindergartens, three kindergartens in each region were drawn at random and approached to participate in the project. Due to the impact of the COVID-19 pandemic which significantly affected the functioning of kindergartens, a large number of kindergartens refused to participate because they did not have any extra capacity to deal with all of the impacts of the pandemic measures. Therefore, additional kindergartens from the list of eligible kindergartens were approached in turn, particularly those that had been willing to participate. The selection respected the proportional representation of the types of kindergartens in the Czech Republic. The study included all children in the last compulsory year in these kindergartens (2019/2020).

This resulted in 67 kindergartens from 11 regions in the Czech Republic with 155 teachers participating in the study. Each teacher thus assessed an average of 5.7 children. In terms of the population of children, a total of 994 children in the last compulsory year of preschool education were involved in the study. One hundred thirty-three children were excluded due to incomplete or erroneous data. The final sample consisted of 861 children, divided into four groups according to the type of kindergarten attended.

### Methods

Attitudes toward school and education were assessed by means of the modified version of the questionnaire used in the ISEP project (MacBeath and Mortimore, 1994; Smees et al., 2002) adapted for use in preschool education. Regarding the use of the questionnaire in a different socio-cultural setting, we tested the characteristics of the questionnaire and found it to be inappropriate in its original form (especially due to low internal consistency and unclear factor structure). A shortened version of the questionnaire consisting of the original 12 items was obtained through a sequential internal consistency analysis, exploratory factor analysis and explanation of the factors found. This version is characterized by a reasonable internal consistency and a well-interpretable three-factor structure (Behavior at school, Feelings toward school, Relationship to educational activities). The questionnaire used a four-point Likert scale. The subsequent analysis used the mean score. The overall Cronbach’s alpha of the modified version of the questionnaire achieved 0.851. Higher scores suggest a higher level in the sense of a positive child’s attitude to school and education.

School performance motivation was assessed by means of the standardized questionnaire School Performance Motivation developed by Hrabal and Pavelková (2011) which measures pupils’ school performance motivation. This 12-item questionnaire was adapted for use among children in the last year of preschool education and used the form of the teacher rating measure on the five-point Likert scale. This performance motivation questionnaire consists of two subscales, the need to achieve school success (total score of 6 items) and the need to avoid school failure (total score of 6 items). The psychometric properties of the adapted version of the questionnaire such as internal scale consistency, relationship properties between the items and total score (item-total statistics) and factor load of the two-factor structure (generalized least squares, factor rotation, Oblimin) were verified and compared with the original version and show a satisfactory validity. Cronbach’s alpha for both scales of the questionnaire shows a very good internal consistency of 0.87 and 0.84, respectively. Higher values indicate a higher level of motivation.

Executive functions were assessed by means of the standardized BRIEF (Behavior Rating Inventory of Executive Function) developed by Gioia et al. (2000) which assesses children’s capacity in coping with common social situations and their overall adaptation abilities. The questionnaire allows the assessment and evaluation of executive functions of children from 5 years of age by means of the teacher’s report/teacher rating measures. The Brief Teacher Form includes 86 items in eight scales which assess the different aspects of executive functions. The results are three cumulative scales: The Behavioral Regulation Index (BRI) captures the child’s ability to regulate and monitor behavior effectively. The Metacognition index (MI) is interpreted as the ability of cognitive task coping and the ability to control one’s behavior. The last cumulative scale is the Global Executive Composite (GEC), which provides the total score of all scales (Ptáček, 2011). The BRIEF shows satisfactory internal consistency, test-retest reliability, moderate inter-rater agreement, and appropriate evidence of predictive and discriminant validity (St John et al., 2018). A higher BRIEF scale score indicates a higher level of executive dysfunction. In this study we used the raw scale score which was transformed to T-score with respect to children’s age and gender. T-scores at or below 59 are considered to be within the typical range. T-scores of 60–64 are in the mildly elevated range, and scores equal to or exceeding 65 are considered to be significantly elevated. In addition to the three cumulative scales, the results also included additional three scales, such as the Working Memory Scale (WM) which assesses the capability to encode information and to hold them when completing a task. The Inhibition Scale refers to the trouble a child can have to control his/her impulses and behavior while the Attention Shift Scale indicates the problems a child can have with moving from one activity to another and/or with flexibly shifting attention (Dekker et al., 2017). In previous studies, these constructs showed positive relationships with school results, whether it be working memory in relation to language pre-literacy (Clark et al., 2010), inhibit control in relation to math performance (Davidson et al., 2006; Gerst et al., 2017) or shifting in relation to math skills (van den Bos et al., 2013).

### Ethics Statement

Signed informed consents were obtained from all of the parents or guardians of the children participating in the study in accordance with the Declaration of Helsinki. The Research Ethics Committee of the Faculty of Education, Palacký University Olomouc, Czech Republic approved the protocol of this study considering all of the ethical aspects of data collection and processing.

### Data Collection

Data collection was performed at the end of the last compulsory year of preschool education from June to August 2020. Data collection was performed in an electronic way using Google forms, which met the methodological and research criteria of online research relevance (e.g., high degree of security, archiving, and encoding during data transfer, access via generated ID). The data were collected through a teacher report. All of the participating teachers were thoroughly and in detail informed about the procedure for administering the questionnaires and had the opportunity to contact the research team at any time in case of any uncertainties or problems. In addition, each questionnaire was provided with very detailed instructions for administration.

### Statistical Analysis

Descriptive statistics were performed on demographic and observed variables. Few missing or inconsistent values were detected. No imputation of missing values was performed, cases with objectionable data were excluded from the related analysis. Due to the differences in the number of participants in the respective groups and the small number of children from religious kindergartens, non-parametric statistical tests were used. The differences in the investigated variables between the individual types of kindergartens were examined using the Kruskal-Wallis test and the Wilcoxon-Mann-Whitney test with the Holm-Bonferroni correction. Finally, eta-squared was calculated to assess the effect size. Any 2-sided *P* < 0.05 was considered statistically significant. Statistical analyses and data visualizations were performed using SPSS 25 (IBM corp., Armonk, NY, United States) and RStudio (v.1.4.1717, RStudio Inc.; R version 4.1.1, The R Foundation for Statistical Computing).

## Results

### Demographics of Preschool Children Sample

The study population consisted of 861 preschool children, of whom 445 (51.7%) were boys and 416 (48.3%) were girls, with a mean age of 6.0 (SD = 0.44). The majority of children (84.9%) attended a traditional kindergarten, with the remaining children (15.1%) attending one of three types of alternative kindergartens (Table 1).

**TABLE 1:** Sample characteristics.

	Data
N	861
**Sex (N [%])**	
Boys	445 [51.7]
Girls	416 [48.3]
**Age (mean ± SD)**	
Sample	6.0 ± 0.44
Boys	6.1 ± 0.46
Girls	5.9 ± 0.40
**Age at entry to kindergarten (mean ± SD)**	
Sample	3.12 ± 0.82
Boys	3.18 ± 0.84
Girls	3.06 ± 0.80
**Type of kindergarten (N [%])**	
Traditional	731 [84.9]
Religious	65 [7.5]
Montessori	35 [4.1]
Waldorf	30 [3.5]

### School Performance Motivation

**FIGURE 1:**
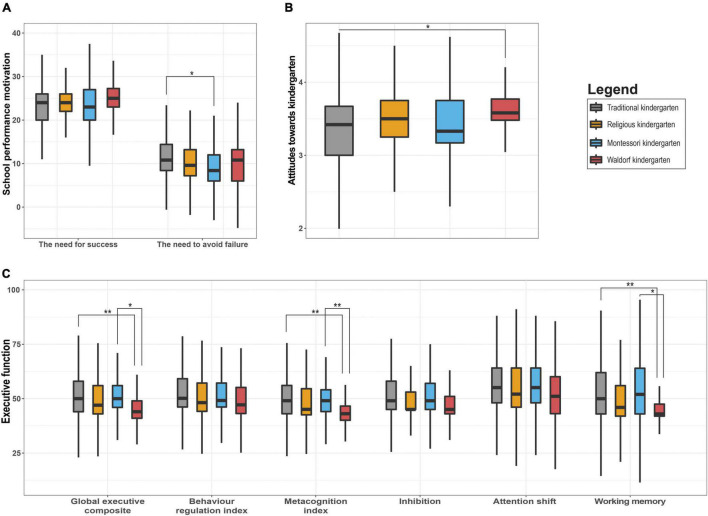
Differences in school performance motivation **(A)**, attitudes toward kindergarten **(B)**, and executive functions **(C)** in different types of kindergartens. The boxplots show the differences in the medians and IQRs of children from individual kindergarten types. The horizontal upper bars represent the differences between individual types of kindergartens with indicated levels of significance (*P < 0.05, **P < 0.01).

First, we examined whether children’s need to succeed and their need to avoid failure differed depending on the type of kindergarten. In general, the need for success appeared to be higher than the need to avoid failure (Table 2). In terms of the differences between kindergartens, although the distribution of values between kindergartens indicated some trends of differences, particularly for the need to avoid failure (Figure 1A), significant differences with a small effect size emerged only between traditional schools and Montessori kindergartens (*P* = 0.024, η^2^ = 0.01), with children from Montessori kindergartens showing lower levels of the need to avoid failure (these children generally showed the lowest need to avoid failure of all kindergarten types) (Table 3).

**TABLE 2:** Description of the dependent variables in the individual types of kindergartens.

	Traditional KDG	Religious KDG	Montessori KDG	Waldorf KDG
N (%)	731 (84.9)	65 (7.5)	35 (4.1)	30 (3.5)
**School performance motivation**				
The need for success	24.0 [20.0–26.0]	24.0 [22.0–26.0]	23.0 [20.0–27.0]	25.0 [23.0–27.3]
The need to avoid failure	10.8 [8.4–14.4]	9.6 [7.2–13.2]	8.4 [6.0–12.0]	10.8 [6.0–13.2]
**Children’s attitudes toward kindergarten**	3.4 [3.0–3.7]	3.5 [3.3–3.8]	3.3 [3.2–3.8]	3.9 [3.5–3.8]
N	713	65	34	29
**Executive functions^a^**				
Global executive composite	50 [44–58]	47 [43–56]	50 [46–56]	44 [41–49]
Behavior regulation index	50 [46–59]	48 [44–57]	49 [46–57]	47 [43–55]
Metacognition index	49 [43–56]	45 [42.5–54.5]	49 [44–54]	43 [40–46.5]
Inhibition	49 [45–58]	45 [45–53]	49 [45–57]	45 [43–51]
Attention shift	55 [48–64]	52 [46–64]	55 [48–64]	51 [43–60]
Working memory	50 [43–62]	46 [42–56]	52 [43–64]	43 [42–47.5]

*KDG, kindergarten.*

*^a^Values are given as T-scores.*

*Values presented as median [IQR].*

**TABLE 3:** Differences in school achievement, motivation and attitudes toward kindergarten.

	Kruskal-Wallis test			Mann-Whitney test (Holm-Bonf. corr.)		
		
	H	P	Eta2	U	P	Eta2
**School performance motivation**						
The need for success	6.049	0.11	0.01			
Traditional KDG ∼ Religious KDG				21,272.5	0.644	<0.01
Traditional KDG ∼ Montessori KDG				12,765.5	0.983	<0.01
Traditional KDG ∼ Waldorf KDG				8524.5	0.228	0.01
Religious KDG ∼ Montessori KDG				992.5	0.879	0.01
Religious KDG ∼ Waldorf KDG				856.5	0.879	0.01
Montessori KDG ∼ Waldorf KDG				402.0	0.520	0.04
The need to avoid failure	12.740	0.005**	0.01			
Traditional KDG ∼ Religious KDG				20,256.0	0.240	<0.01
Traditional KDG ∼ Montessori KDG				9105.5	0.024*	0.01
Traditional KDG ∼ Waldorf KDG				9479.5	0.824	<0.01
Religious KDG ∼ Montessori KDG				965.0	0.824	0.02
Religious KDG ∼ Waldorf KDG				972.5	0.984	<0.01
Montessori KDG ∼ Waldorf KDG				456.5	0.824	0.01
**Children**’**s attitudes toward kindergarten**	13.430	0.004**	0.02			
Traditional KDG ∼ Religious KDG				19,899.0	0.120	0.01
Traditional KDG ∼ Montessori KDG				12,328.5	0.716	<0.01
Traditional KDG ∼ Waldorf KDG				7379.5	0.012**	0.01
Religious KDG ∼ Montessori KDG				975.0	0.579	0.01
Religious KDG ∼ Waldorf KDG				813.0	0.579	0.02
Montessori KDG ∼ Waldorf KDG				351.5	0.110	0.08

*KDG, kindergarten.*

*P < 0.05, **P < 0.01.

### Children’s Attitudes Toward Kindergarten

Next, we searched for differences in children’s attitudes toward the kindergartens. The findings showed again a relatively similar level of positive perception of kindergartens by children with traditional and Montessori kindergartens showing the lowest level of positive attitude and Waldorf kindergartens having the highest score (Table 2). A significant difference with a small effect size emerged only between traditional and Waldorf kindergartens (with a difference between Montessori and Waldorf kindergartens being non-significant most likely due to the small size of both groups), with Waldorf kindergartens showing more positive scores (*P* = 0.012, η^2^ = 0.01) (Figure 1B and Table 3). The differences between the other kindergarten types were again non-significant.

### Executive Functions

Finally, we investigated the differences in problems in executive functions depending on the type of kindergarten. In terms of the interpretation of the T-score in each type of kindergarten, the findings revealed that three quarters of children were in the normal range (*T*-score < 60) (Table 2). Comparison-wise, the results showed significant differences in the Global executive composite score (GEC), Metacognition index (MI), and Working memory (WM) (Figure 1C and Table 4). In all of the cases, two different pairs of kindergartens were identified: children from Waldorf kindergartens showed significantly better scores compared with traditional (P_GEC_ = 0.006, η^2^_GEC_ = 0.02, P_MI_ = 0.003, η^2^_MI_ = 0.02, P_WM_ = 0.001, η^2^_WM_ = 0.02) and Montessori (P_GEC_ = 0.020, η^2^_GEC_ = 0.13, P_MI_ = 0.005, η^2^_MI_ = 0.17, P_WM_ = 0.025, η^2^_WM_ = 0.12) kindergartens. Effect sizes between Waldorf and traditional kindergartens were small, while between Montessori and Waldorf they were moderate for Working memory and large for global Executive composite and Metacognition index. Traditional and Montessori kindergartens had similar scores in all of the dimensions of executive functions, religious kindergartens were closer toward Waldorf ones. No significant differences were found within religious kindergartens in relation to other kindergartens.

**TABLE 4:** Differences in executive functions between kindergartens.

	Kruskal-Wallis test			Mann-Whitney test (Holm-Bonf. corr.)		
	**H**	**P**	**Eta2**	**U**	**P**	**Eta2**
		
**Executive functions**						
Global executive composite	14.694	0.002**	0.02			
Traditional KDG ∼ Religious KDG				19,958.0	0.192	0.01
Traditional KDG ∼ Montessori KDG				11,969.5	0.902	<0.01
Traditional KDG ∼ Waldorf KDG				6,492.0	0.006**	0.02
Religious KDG ∼ Montessori KDG				924.5	0.366	0.02
Religious KDG ∼ Waldorf KDG				697.0	0.176	0.04
Montessori KDG ∼ Waldorf KDG				285.5	0.020*	0.13
Behavior regulation index	6.886	0.076	0.01			
Traditional KDG ∼ Religious KDG				20,811.0	0.660	<0.01
Traditional KDG ∼ Montessori KDG				11,290.0	0.250	<0.01
Traditional KDG ∼ Waldorf KDG				7,802.5	0.150	0.01
Religious KDG ∼ Montessori KDG				1,055.5	0.715	<0.01
Religious KDG ∼ Waldorf KDG				797.5	0.660	0.01
Montessori KDG ∼ Waldorf KDG				392.5	0.660	0.03
Metacognition index	17.022	0.001**	0.02			
Traditional KDG ∼ Religious KDG				20,173.5	0.249	<0.01
Traditional KDG ∼ Montessori KDG				11,492.0	0.609	<0.01
Traditional KDG ∼ Waldorf KDG				6,120.0	0.003**	0.02
Religious KDG ∼ Montessori KDG				885.5	0.249	0.03
Religious KDG ∼ Waldorf KDG				665.5	0.092	0.05
Montessori KDG ∼ Waldorf KDG				252.5	0.005**	0.17
Inhibition	10.531	0.015**	0.01			
Traditional KDG ∼ Religious KDG				19,219.0	0.110	0.01
Traditional KDG ∼ Montessori KDG				11,774.0	0.776	<0.01
Traditional KDG ∼ Waldorf KDG				7,628.0	0.096	0.01
Religious KDG ∼ Montessori KDG				942.5	0.672	0.01
Religious KDG ∼ Waldorf KDG				828.5	0.680	0.01
Montessori KDG ∼ Waldorf KDG				380.5	0.468	0.04
Attention shift	4.516	0.211	0.01			
Traditional KDG ∼ Religious KDG				21,422.0	0.999	<0.01
Traditional KDG ∼ Montessori KDG				11,879.0	0.999	<0.01
Traditional KDG ∼ Waldorf KDG				8,167.0	0.324	0.01
Religious KDG ∼ Montessori KDG				1041.0	0.999	<0.01
Religious KDG ∼ Waldorf KDG				822.0	0.999	0.01
Montessori KDG ∼ Waldorf KDG				396.5	0.910	0.03
Working memory	17.081	0.001**	0.02			
Traditional KDG ∼ Religious KDG				19,262.0	0.096	0.01
Traditional KDG ∼ Montessori KDG				11,810.0	0.800	<0.01
Traditional KDG ∼ Waldorf KDG				6,329.5	0.002**	0.02
Religious KDG ∼ Montessori KDG				887.0	0.315	0.03
Religious KDG ∼ Waldorf KDG				764.5	0.315	0.02
Montessori KDG ∼ Waldorf KDG				292.5	0.025*	0.12

*KDG, kindergarten.*

*P < 0.05, **P < 0.01.

## Discussion

The main aim of this study was to analyze the differences in a series of domains related to future school achievement for children in different types of kindergartens.

This study examined several characteristics of preschool children that are related to later school achievement. The first area was school or preschool performance motivation. The results revealed that the need to succeed, rather than the need to avoid failure, could be observed in children. This result is in line with the general characteristic of this developmental period, where the child’s growing need for discovery and exploration gradually comes to the fore, and at the same time, preschool education satiates this need for successful mastery of new skills and knowledge, given that the kindergarten environment principally fosters (or should foster) this curiosity (North American Association for Environmental Education [NAAEE], 2016) and attempts to give it a more formalized character, and yet does not include a strong evaluative element of the child’s success in this endeavor. Thus, the need to avoid failure that might limit the child’s exploratory efforts is not yet fully formed at this level of education within the framework of self-regulated behavior management.

In the context of comparing the different types of kindergartens, the findings on both dimensions of performance motivation were almost equal, suggesting that the impact of the different preschool educational approaches is very similar despite the different education methods. However, the revealed overall lower scores (although non-significant except for the comparison with traditional kindergartens) of the need to avoid failure in Montessori kindergarten children also indicates a trend that the strong element of a non-authoritative, non-coercive education style without the absence of assessment, together with the natural support for success and the child’s own self-esteem (Marshall, 2017) which is typical of this educational philosophy shifts the balance in the individual elements of performance motivation more in favor of the positive need for success. This motivational shift is one of the factors that translates into better school performance, executive function levels and overall performance of these children later in their education (Miller and Bizzell, 1984; Széll, 2013; Lillard et al., 2017; Pholphirul, 2017; Denervaud et al., 2019; Puhan et al., 2019; Dorji et al., 2020; Carulla et al., 2021).

The second investigated factor was children’s attitudes toward kindergarten. The results showed generally positive attitudes of children toward kindergarten and only relatively small differences between the children from different types. One of the main reasons for this positive attitude is probably that kindergartens, regardless of educational philosophy, help to nourish and develop the needs children naturally have during this developmental period. Not only do they enable them to acquire a wide range of new knowledge, but they also create a social environment where children can meet a diverse group of other children, in which they fulfill their need for social interaction and build the foundations of wider social skills. Encouraged by the supportive approach of the teachers, this attitude toward kindergarten is the basis for future attitudes toward education in general and thus, in a broader perspective, influences future motivation for school achievement (Fuhs et al., 2013; Stephanou, 2014; Daviran, 2015). This effect is even more important for children from disadvantaged or unstimulating backgrounds, where kindergarten can play an important role in eliminating these deficits (Currie, 2001; Duncan et al., 2007; Camilli et al., 2010).

The most positive attitude of the children appeared to be toward the Waldorf kindergarten, which was significantly more favorable than that of the traditional kindergartens (with a certain trend in difference being also observable in the kindergarten). The main reasons for this positive perception of Waldorf may be the less structured and teacher-directed classroom environment (Valeski and Stipek, 2001) and the overall relaxed and supportive style of education, the promotion of movement (which is usually an enjoyable and desirable activity for children), the emphasis on teacher-child relationships and the adaptation of the pace to the child’s current development (along with other characteristics of this type of education), which creates a more pleasant environment for the child compared to other types of kindergartens (Sobo, 2015). A surprising result in this context is the relatively lower rating of the Montessori kindergarten, whose characteristics of the environment and way of functioning are closer to Waldorf kindergarten than to the other types. Given the relatively small number of children in the sample, this attitude may be due to the individual characteristics of the children in the study, influenced by the current perceptions of the evaluating teacher, or due to some other factor not captured by this study. This assumption is supported by the fact that, given the developmental capabilities of preschool children and the form of the attitude survey (teacher-rating), these results are primarily a reflection of children’s outward (manifest) attitudes toward kindergarten, both in the emotional and behavioral components.

Finally, we analyzed the differences in potential problems in executive functions. Proper development of executive functions already in the preschool period (and further on during school attendance) is an important element of the educational process for two main reasons. First, executive functions contribute significantly to later school achievement, for example in the context of literacy and mathematics (Blair and Razza, 2007; Jongejan et al., 2007; van den Bos et al., 2013; Becker et al., 2014; Bexkens et al., 2015; Dekker et al., 2017). And second, they are in more general way one of the key mechanisms related to human life functioning. A comparison of the study results with the rating guidelines of the BRIEF test showed in general an absence of significant difficulties in executive functions in all groups of children. T-scores were below the 60-point threshold (which indicates a mildly elevated risk of executive function difficulties) in three-quarters of the children, with the remaining quarter showing signs of mildly elevated risk. Slightly higher values were seen only within Attention shift and for some types of kindergarten for working memory, but again the third quartile was around the above-mentioned threshold and the median was well below this level.

Comparison-wise, the results showed differences in three dimensions: Global executive composite, Metacognition index and Working memory, with all showing better scores for Waldorf kindergartens compared with the other types (with the same trend indicated for the other dimensions of executive functions). The Global Executive Composite represents the aggregate score of all scales and its values are thus a summary of the levels in each dimension of executive function. The Metacognition index describes the ability to cognitively self-manage tasks and to monitor one’s own performance and includes working memory as a subscale. Although it might be reasoned that education incorporating clearly defined rules and a variety of stimuli would be more appropriate for the development of individual skills, the better results of Waldorf schools suggest that approach that favors the child’s development at his or her own pace without a strong external order and organization of activities is a more appropriate setting. Given the lower self-regulatory abilities of children at this age and the still developing attentional control which is an essential mechanism of working memory (Oberauer, 2019), this approach seems to encourage more internal drive to develop and create better (more stable) conditions for effective development of individual executive functions. This motivation may be further saturated by an overall positive perception of the kindergarten environment, as well as external influences, especially parental support, and perceptions of the kindergarten (Pandey and Thapa, 2017; Casey et al., 2018; McDowell et al., 2018).

In the same context, traditional and Montessori kindergartens showed significantly lower scores as compared to Waldorf kindergarten. Particularly in the context of Montessori kindergartens, this is again a surprising finding, as the nature of the work with children supports rather similar expectations of outcomes to those of Waldorf kindergartens, as reported in previous studies (Cox and Rowlands, 2000; Lillard, 2006, 2012; Murray, 2010; Ansari and Winsler, 2014; Denervaud et al., 2019). Thus, this deviation requires further investigation to uncover the reasons or to eliminate any bias associated with the current research sample. In the observed group of kindergartens with alternative elements of education, there were no statistically significant results concerning religious kindergartens. On the basis of these results, it is impossible to clearly define which factors may have influenced these findings. Further research could proceed in this direction.

### Limitations

This study has a few limitations. The first is the relatively low number of children for each type of alternative kindergarten and their disproportionality to respondents in traditional kindergartens. In fact, the low numbers of respondents in the groups may affect the evaluation of the significance of the differences between the groups, and the negative effect of outliers may skew the scores of the entire group. However, the actual proportion of respondents within each kindergarten is broadly consistent with the actual distribution of children within preschool education.

The second limitation is potential rater bias. Given that the methods used were teacher report (as is standard in research with this population), the findings obtained (especially in the context of the perceptions of the kindergarten environment and children’s performance motivation) may be influenced to some extent by the teacher’s own perception of the type of kindergarten, its philosophy, and of the children themselves and their characteristics. We can hypothesize (also due to the imbalance in the number of different types of kindergartens in the education system) that the choice to work in a particular type of kindergarten is linked to an internal congruence with the educational philosophy of the kindergarten, which may lead to a certain overestimation of the quality of the educational process in the kindergarten and a more positive perception of children and their abilities and attitudes.

Despite the above limitations, the study provides an important and differentiated description of the factors that may affect future school performance. In order to deepen this description, future research should examine other important factors that significantly influence the child’s attitudes and readiness for school, among which the family’s contribution is paramount.

Although undesirable, the socio-economic status of the family still remains the strongest predictor of school achievement within the Czech education system. Therefore, it would be important to deeply analyze its impact on the results of kindergarten education. It would be especially beneficial to study the parents’ attitudes toward the choice of kindergarten and school curriculum. Their parenting practices also have a significant impact on their child’s motivation and social and cognitive development. Last but not least, it is also the degree of agreement between the educational practices of parents and kindergarten that can moderate the impact of the educational benefits of kindergarten.

A further direction for future research should provide a deeper understanding of the effectiveness of educational styles and practices in different types of kindergartens. This will require longitudinal research into the benefits of schooling. This type of research could also shed light on the necessary organizational, systematic and educational conditions that are necessary for children in kindergartens to fully develop their potential.

## Conclusion

This study provides a relatively scarce comparison of the factors that can influence future school achievement in a comprehensive set of different types of traditional and alternative kindergartens. In conclusion, the findings showed only minimal differences in the measured predictors of later school achievement (children’s attitudes toward kindergarten, executive functions, school performance motivation) between the different types of kindergartens in the Czech Republic. Irrespective of the initial philosophy of the approach to preschool education and the different way of conducting education, children’s development is very similar. At the same time, different types of kindergartens have their own specific strengths. Montessori education is perceived most positively by children and promotes achievement-oriented executive motivation, while the Waldorf approach is characterized by the best results in terms of executive development. Thus, although all types of kindergartens included in our study are perceived positively by children and develop children sufficiently in all areas related to future executive motivation, alternative preschool education appears to be more successful in preparing children for the future.

## Data Availability Statement

The datasets presented in this article are not readily available because informed consent does not cover the sharing of data to a third party. Requests to access the datasets should be directed to JK, jana.kvintova@upol.cz.

## Ethics Statement

The studies involving human participants were reviewed and approved by the Research Ethics Committee of the Faculty of Education, Palacký University Olomouc, Czechia. Written informed consent to participate in this study was provided by the participants’ legal guardian/next of kin.

## Author Contributions

JK conceived the idea of this study. RC performed the statistical analysis. JK, LK, and RC wrote the draft of the manuscript. All authors reviewed and contributed to the critical revision of the manuscript for important intellectual content and participated in data collection.

## Conflict of Interest

The authors declare that the research was conducted in the absence of any commercial or financial relationships that could be construed as a potential conflict of interest.

## Publisher’s Note

All claims expressed in this article are solely those of the authors and do not necessarily represent those of their affiliated organizations, or those of the publisher, the editors and the reviewers. Any product that may be evaluated in this article, or claim that may be made by its manufacturer, is not guaranteed or endorsed by the publisher.
